# Fluorescent-increase kinetics of different fluorescent reporters used for qPCR depend on monitoring chemistry, targeted sequence, type of DNA input and PCR efficiency

**DOI:** 10.1007/s00604-013-1155-8

**Published:** 2014-01-14

**Authors:** Jan M. Ruijter, Peter Lorenz, Jari M. Tuomi, Michael Hecker, Maurice J. B. van den Hoff

**Affiliations:** 1 Department of Anatomy, Embryology & Physiology, Academic Medical Center, Meibergdreef 15, 1105AZ Amsterdam, The Netherlands; 2 Institute of Immunology, University of Rostock, Rostock, Germany; 3 Northern Ontario School of Medicine, Sault Ste. Marie, ON Canada; 4 Steinbeis Transfer Center for Proteome Analysis, Rostock, Germany

**Keywords:** Quantitative PCR, Monitoring chemistry, DNA-binding dyes, Hydrolysis probes, Hybridization probes, PCR efficiency

## Abstract

The analysis of quantitative PCR data usually does not take into account the fact that the increase in fluorescence depends on the monitoring chemistry, the input of ds-DNA or ss-cDNA, and the directionality of the targeting of probes or primers. The monitoring chemistries currently available can be categorized into six groups: (A) DNA-binding dyes; (B) hybridization probes; (C) hydrolysis probes; (D) LUX primers; (E) hairpin primers; and (F) the QZyme system. We have determined the kinetics of the increase in fluorescence for each of these groups with respect to the input of both ds-DNA and ss-cDNA. For the latter, we also evaluated mRNA and cDNA targeting probes or primers. This analysis revealed three situations. Hydrolysis probes and LUX primers, compared to DNA-binding dyes, do not require a correction of the observed quantification cycle. Hybridization probes and hairpin primers require a correction of −1 cycle (dubbed C-lag), while the QZyme system requires the C-lag correction and an efficiency-dependent C-shift correction. A PCR efficiency value can be derived from the relative increase in fluorescence in the exponential phase of the amplification curve for all monitoring chemistries. In case of hydrolysis probes, LUX primers and hairpin primers, however, this should be performed after cycle 12, and for the QZyme system after cycle 19, to keep the overestimation of the PCR efficiency below 0.5 %.

**Electronic supplementary material:**

The online version of this article (doi:10.1007/s00604-013-1155-8) contains supplementary material, which is available to authorized users.

**Figure:**
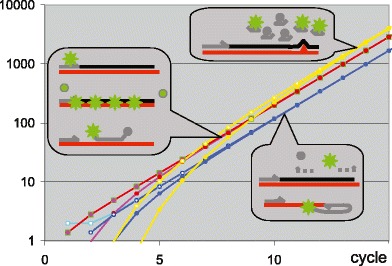
The qPCR monitoring chemistries form six groups with distinct fluorescence kinetics. The displacement of the amplification curve depends on the chemistry, DNA input and probe-targeting. The observed shift in C_q_ values can be corrected and PCR efficiencies can be derived.

## Introduction

**Fig. 1:**
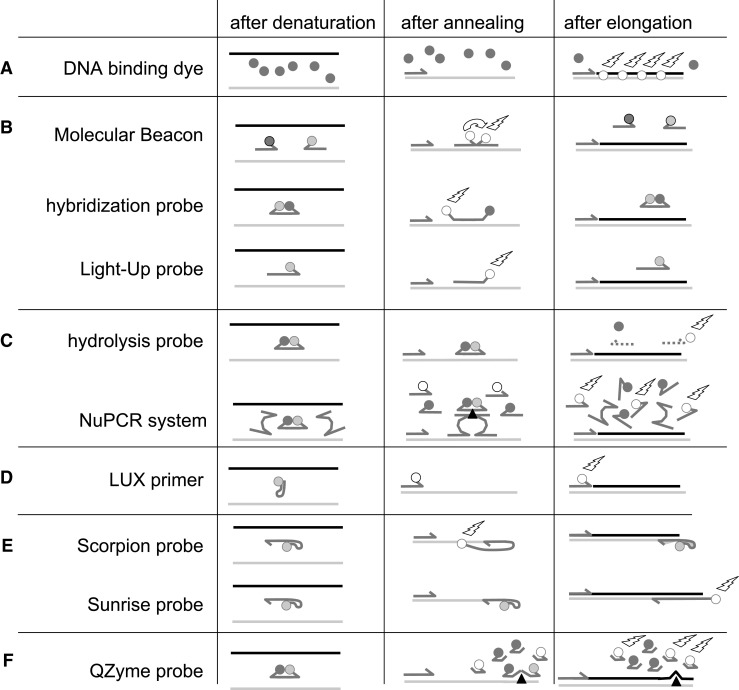
Comparison of the various monitoring chemistries discussed in this paper. For each chemistry group (**a**–**f**), the fluorescence status of the reporter or probe is illustrated after denaturation, annealing and elongation. Hybridization probes, Molecular Beacons and Light-Up probes have been placed together in group B because they lead to equivalent fluorescence increase in all situations. For the same reason, hydrolysis probes and NuPCR are combined in group C and Scorpion and Sunrise probes in group E. *Light grey strands* represent the cDNA strand, *black strands* are synthesized complementary strands, primers and probes are shown as *dark grey half arrows. Dark grey circles* are quenchers; *light grey circles* are quenched fluorophores whereas white circles with lightning flashes represent observed fluorescence emission. *Black triangles* indicate DNAzyme activity

Monitoring the increasing amount of PCR product with fluorescent reporters has enabled the evolution of quantitative PCR (qPCR) into the method of choice for measuring small amounts of DNA or RNA [1,2]. A large variety of monitoring chemistries is currently available for this purpose [3] (Fig. 1 and Fig. S1 (Electronic Supplementary Material, ESM)). The amplification curve thus shows the amount of fluorescence which exponentially increases during cycling. The analysis of qPCR data, however, is mainly based on an equation that relates the position of this curve to the original target concentration [4]. Virtually all qPCR data analyses thus rely on adaptations and simplifications of the inverse of the basic kinetic equation of the polymerase chain reaction [5]. In this inverse equation, the fractional number of cycles (quantification cycle C_q_) that is needed to reach a set amount of fluorescence (quantification threshold Nq) and the PCR efficiency (E) are used to calculate the starting concentration, or target quantity, of the DNA fragment of interest (N_0_). This calculation does not take into account that the kinetics of fluorescence increase depend on the monitoring chemistry or that these kinetics depend on whether the qPCR cycling starts with ss-cDNA or ds-DNA. Moreover, probe sequences may be designed to target the cDNA sequence or the mRNA sequence and the linking of adapters may be part of the monitoring chemistry, resulting in differences in fluorescence release in the first PCR cycle(s) that have lasting effects in later cycles.

It was reported that an efficiency-dependent bias occurs in C_q_ values when the monitoring fluorescence increases cumulatively [6]. However, in that paper only monitoring with DNA-binding dyes and hydrolysis probes were considered and the effects of differences in input, i.e. ss-cDNA or ds-DNA, and probe-targeting were not taken into account. The current paper aims to complete the picture by distinguishing six groups of monitoring chemistries for qPCR. For each chemistry, the fluorescence kinetics and its C_q_ bias for input of ss-cDNA or ds-DNA, as well as for sense and anti-sense probing, were determined and the C_q_ correction was specified. This correction fully eliminates the C_q_ biases. The PCR efficiency value can be correctly derived from the individual amplification curves for all chemistries.

## Definitions

To avoid confusion the following definitions will be used throughout this paper. The mRNA sequence will be considered to be the sense sequence; consequently the first-strand cDNA sequence (ss-cDNA) is anti-sense. A probe, or primer, targeting the mRNA sequence is thus an anti-sense probe (also reverse primer); a probe targeting the cDNA sequence is a sense probe (also forward primer). One PCR cycle consists of three phases: denaturation, annealing, and elongation; a cycle is considered to start with the denaturation phase. PCR efficiency (E) is defined as the fold increase of the amount of DNA per cycle and has a theoretical maximum of 2 (=100 %) corresponding to a doubling of input material in each cycle.

C_shift_ was defined as the efficiency-dependent factor that has to be added to the observed C_q_ to correct the leftward horizontal shift in the C_q_ value due to fluorescence accumulation [6]. A second correction factor, dubbed C_lag_, will be introduced to correct for fluorescence series that start with one or more cycles without amplification-dependent fluorescence.

The amount of fluorescence observed, or released, when one molecule of ds-DNA amplicon is created, is defined as 1 unit of reporter fluorescence.

## Experimental

### Method

**Fig. 2:**
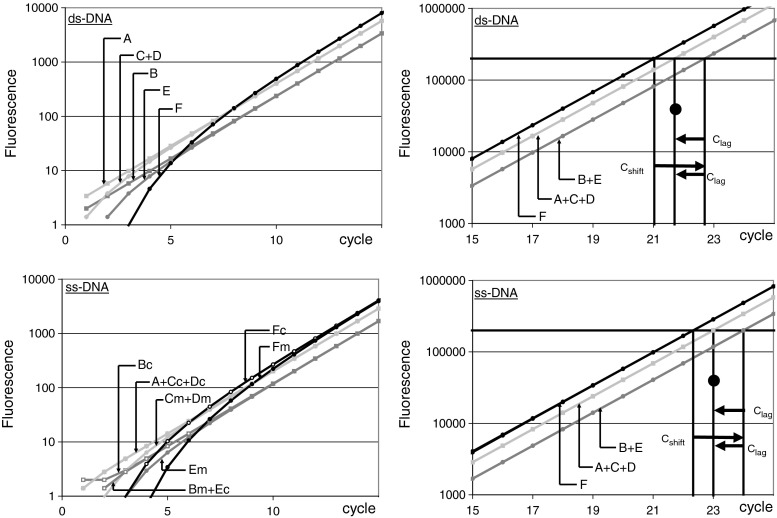
Amplification curves for all six chemistries were simulated for PCR efficiency of 1.7 and input of ds-DNA (*top*) and ss-cDNA (*bottom*) plotted on a logarithmic fluorescence axis. Chemistry groups are identified by the character that was assigned in Fig. 1 with the suffixes ‘c’ and ‘m’ to indicate targeting of the cDNA or mRNA sequence for ss-cDNA input. On the left, the first 20 cycles are shown to illustrate the differences in initial increase of fluorescence between chemistries. Despite those initial differences, the amplification curves converge into groups of overlapping parallel straight lines for all PCR efficiencies. On the right the cycles around the C_q_ values are shown. Note that depending on the chemistry the C_q_ values are shifted with respect to the C_q_ that is observed with a DNA-binding dye (*black circle*). Chemistries in groups A, C and D do not require a C_q_ correction (●); chemistries in groups B and E require a C_lag_ correction (←) and the chemistry in group F requires a C_shift_ correction (→) and a C_lag_ (←) correction (See Figs. S8 and S9, ESM, for amplification curves with PCR efficiency of 2)

Different monitoring chemistries were extracted from publications and manufacturer information, and their mode of fluorescence increase during PCR cycling was determined (Fig. 1; Figs. S1–S7, ESM). Based on these results, the fluorescence increase during cycling was simulated in Microsoft Excel for different PCR efficiency values (Fig. 2; Supplementary Excel file, ESM). In this simulation, the number of DNA strands originating from the input strand at the given PCR efficiency value is determined for each cycle and the fluorescence associated with each strand reported. Because the simulation aimed at the direct effect of the monitoring chemistry and DNA-targeting on the observed C_q_ value, no random noise was included in the calculations. After setting a quantification threshold and determining the quantification cycle (C_q_), the bias in the resulting target concentrations could be determined (Fig. 2). From this the required C_q_ correction could be derived.

Note that the absolute amount of fluorescence associated with 1 unit of reporter differs between chemistries. However, this does not affect the simulation and the results of this analysis because analysis of the qPCR amplification curves is based on the relative change in fluorescence from cycle to cycle within each PCR reaction [4]. In other words, the amount of fluorescence observed per unit generated DNA affects the scale of the fluorescence axis but not the derived PCR efficiency and C_q_ value.

### Results

#### Basic principles

Comparison of the various chemistries reveals a number of principles that characterize the fluorescence appearance during monitoring of the qPCR cycles.

1. Fluorescent reporters can be divided into a) DNA-binding compounds, binding ds-DNA (DNA-binding dyes) or ss-DNA (annealing probes: hybridization probes, Molecular Beacons and Light-Up probes), that are released unscathed in the elongation phase and b) compounds that become fluorescent as result of the elongation of the primers, either by removal of quenching through digestion of the probe (hydrolysis probes: TaqMan, QZyme and NuPCR), or through creation of a physical distance between reporter and quencher (LUX primers, Scorpion probes and Sunrise probes).
2. The input can be either ss-cDNA or ds-DNA. In the former, only the anti-sense strand is available for primer or probe annealing in the first cycle, whereas in the latter both strands are available. This difference makes the appearance of fluorescence dependent on the type of input DNA and the targeted strand.
3. The probe sequence is designed to target either of the two DNA strands. When an assay is based on input of ss-cDNA, in the first cycle only the anti-sense (cDNA) sequence is present. The combination of this polarity of the probe and the presence of the targeted sequence in the input DNA will, therefore, determine whether a unit of fluorescence is observed in the first or in the subsequent cycles and is propagated through the entire PCR run.

#### Chemistry groups

Based on these three principles six groups of monitoring chemistries (Fig. 1; Fig. S1, ESM) can be distinguished. For each of those, three different fluorescence kinetics can occur (Table 1; Figs. S2–S7, ESM) depending on the input (ss-cDNA or ds-DNA) and the targeted sequence (sense or anti-sense) when the input is cDNA. In the following description of the different kinetics the PCR efficiency is assumed to be 2 (100 % efficient PCR reaction; Figs. S8 and S9, ESM). Amplification curves resulting from a PCR efficiency value of 1.7 are shown in Fig. 2 (numerical data given in the Supplementary Excel file, ESM). Since the fluorescence signal is generated at a different phase of the PCR cycle for different chemistries, the PCR instrument software has to be adjusted to perform data acquisition in the correct phase.

**Table 1:** The fluorescence-increase kinetics for PCR efficiency of 2 (=100 %) is given for the first five cycles and each chemistry group, DNA input and targeting sequence

		Cycle					C_q_ Correction	
Chemistry Group		1	2	3	4	5	C_lag_	C_shift_
Input: ds-DNA								
A		2	4	8	16	32		
B		1	2	4	8	16	−1	
C		1	3	7	15	31		
D		1	3	7	15	31		
E			1	3	7	15	−1	
F				1	5	16	−1	+ C_shift_
Input: ss-cDNA	Targeted sequence							
A	–	1	2	4	8	16		
B	cDNA	1	1	2	4	8	−1	
	mRNA		1	2	4	8	−1	
C	cDNA	1	2	4	8	16		
	mRNA		1	3	7	15		
D	cDNA	1	2	4	8	16		
	mRNA		1	3	7	15		
E	cDNA		1	2	4	8	−1	
	mRNA			1	3	7	−1	
F	cDNA			1	4	11	−1	+ C_shift_
	mRNA				1	5	−1	+ C_shift_

The correction of the C_q_ value depends on the chemistry and is given in the last two columns. C_lag_ is always −1; C_shift_ depends on the PCR efficiency (see text). To transform the C_q_ values observed for ss-cDNA input to those observed with ds-DNA, for equivalent copy-number, log(2)/log(E) should be subtracted from the C_q_ value

**Group A** consists of the DNA-binding dyes, like SYBR Green, that become fluorescent upon binding to ds-DNA [7] (Fig. 1a). DNA-binding dyes display the standard exponential increase of fluorescence, following the mathematical pattern of 1-2-4-8 when the input is ss-cDNA and 2-4-8-16 when the input is ds-DNA (Fig. S2, ESM).

**Group B** includes annealing probes of which the fluorescence is measured during the annealing phase and comprises three different chemistries that display the same kinetic behavior. (1) Hybridization Probes [8] consist of two fluorescently labeled probes that have to anneal next to each other on the same DNA strand. Due to the close proximity of the two fluorochromes, Fluorescence Resonance Energy Transfer (FRET) occurs (Fig. 1b). (2) Molecular Beacons [9] are primers with a hairpin-loop that keeps the fluorescent reporter and quencher close together; after annealing the hairpin opens up, allowing the reporter to emit fluorescence (Fig. 1b). (3) Light-Up probes are peptide nucleic acid (PNA) oligonucleotides to which a thiazole dye is tethered. Probe annealing allows the dye to interact with the probe-DNA hybrid and to become fluorescent [10] (Fig. 1b). When annealing probes target the cDNA sequence the fluorescence follows a 1-2-4-8 series with ds-DNA as input and a 1-1-2-4-8 series with ss-cDNA input; targeting of the mRNA sequence always results in a 0-1-2-4-8 series (Fig. S3, ESM).

**Group C** comprises the hydrolysis probes [11]. In these probes a reporter and a quencher are in close proximity, ensuring no (or low) fluorescence in both the unbound and annealed state. During elongation the annealed probe is digested by the polymerase and the quenching is abolished (Fig. 1c). The released reporter is, and remains, fluorescent in all subsequent cycles [6]. This group also includes the recently introduced NuPCR [12] which consists of two adjacent probes that bring together two halves of a DNAzyme. This DNAzyme subsequently cleaves a universal hydrolysis probe, releasing its reporter (Fig. 1c). The fluorescence increase of hydrolysis probes follows a 1-3-7-15 series in case of ds-DNA. In case of ss-cDNA input, a 0-1-3-7 series is found when the probe targets the mRNA sequence and a 1-2-4-8 series when the cDNA sequence is targeted (Fig. S4, ESM).

**Group D.** In the Light-Upon-eXtension (LUX) monitoring chemistry [13,14], one of the PCR primers is tagged with a fluorescent reporter within a hairpin structure that quenches the reporter. During annealing the hairpin opens and the reporter becomes fluorescent (Fig. 1d). After elongation the primer becomes incorporated into the amplification product and remains fluorescent. As a consequence fluorescence follows a 1-3-7-15 series when ds-DNA is used as input. In case of ss-cDNA input, the fluorescence follows 1-2-4-8 and 0-1-3-7 series when the cDNA and the mRNA sequences are targeted (Fig. S5, ESM).

**Group E** consists of Scorpion [15] and Sunrise primers [16]. In this chemistry, one primer is tagged with a DNA sequence that forms a secondary structure and quenches the reporter. This quenching is permanently lost in the cycle following the cycle in which the primer is incorporated into the synthesized strand (Fig. 1e). When the reporter-containing primer targets the cDNA sequence and the input is ss-cDNA, the fluorescence increase follows a 0-1-2-4-8 series; targeting the mRNA sequence results in a 0-0-1-3-7 series. In case of ds-DNA input the fluorescence increase follows a 0-1-3-7-15 series (Fig. S6, ESM).

**Group F** consists of the QZyme system [17]. This system comprises two primers and a quenched reporter substrate. One of the primers, the QZyme-primer, is a gene-specific primer tagged with a DNAzyme encoding sequence in its inactive orientation. During the subsequent PCR cycles the complementary strand is synthesized, creating the active QZyme, which binds and cleaves the reporter substrate, releasing the fluorescent reporter (Fig. 1f). Because the DNAzyme is activated in every annealing phase the fluorescence increase is exponential. The fluorescence increase follows a 1-5-16 series in case of ds-DNA and ss-cDNA input and the QZyme primer targets the mRNA sequence; a 1-4-11 series is found when ss-cDNA input is used and the QZyme primer targets the cDNA sequence (Fig. S7, ESM).

Besides the qPCR monitoring chemistries described above, there are other systems on the market. The AmpliFluor system depends on a cascade of four cycles, in which a universal target sequence is linked to the amplicon sequence and a Scorpion-like reporter system is targeting this primer [18]. A fluorescence-increase series can be derived, assuming that the annealing and elongation of all primers is fully efficient and independent of each other. Because we did not consider this to be a realistic scenario, and because the description of AmpliFluor system varied in the available documentation, this monitoring system was excluded.

#### Required C_q_ corrections

Although fluorescent monitoring of the DNA synthesis is based on very different chemistries, and depends on the combination of type of input DNA and the polarity of the reporter-containing primer or probe, only a limited number of different fluorescence increase kinetic series occur. Each of these combinations was simulated in such a way that the starting concentration (N_0_), the PCR efficiency (E) and the quantification threshold (C_q_) could be varied (Supplementary Excel file). The results of these simulations are shown for PCR efficiency 1.7 (Fig. 2). The effect on the observed C_q_ values was determined, as well as the correction required to reach the correct target quantity. The correct quantity is defined as the target quantity that served as input and was observed with a DNA-binding dye. Correction of C_q_ was determined for either ss-cDNA or for ds-DNA input. Note that the C_q_ values after ds-DNA input are always log(2)/log(E) lower than after ss-cDNA input when the same monitoring chemistry is applied.

Although the fluorescence increase differs in the first cycles, on a logarithmic fluorescence axis all curves converge to two groups of superimposed straight lines when the PCR efficiency is set to 2 (Figs. S8 and S9, ESM). For both ss-cDNA and ds-DNA input, this gives the impression that a simple -1 correction of the observed C_q_ values would be sufficient to correct for the delay in fluorescence increase which causes the one cycle right shift of the amplification curve. However, such a correction would not suffice when the PCR efficiency is not 2 because for such lower PCR efficiency values more ‘amplification curves’ appear (Fig. 2). It turns out that, for both ss-cDNA and ds-DNA input, the same two types of correction of the C_q_ values are required to reach the correct target quantity (Fig. 2; Table 1).

Firstly, chemistries in group B, E and F require the subtraction of one cycle, dubbed C_lag_, from the observed C_q_. The reason for this correction differs per chemistry group. For annealing probes (group B) only 1 unit of fluorescence is observed compared to every 2 units that are observed with a DNA-binding dye (Figs. S2 and S3). In group B, C_lag_ thus corrects for the fact that fluorescence increase of annealing probes lags one cycle behind that of DNA-binding dyes throughout cycling. Although hydrolysis probes (group C) suffer the same initial deficit (Fig. S4), the cumulative nature of their fluorescence increase itself already corrects this deficit. However, despite their cumulative nature, the chemistries in group E and F do require C_lag_ correction because their fluorescence, even with ds-DNA input, does not start in the first cycle (Figs. S5 and S6); in these chemistries C_lag_ corrects for this initial lag.

A second correction is required for group F, the QZyme system (Fig. S7, ESM). The repeated reactivation of the DNAzyme that is build into the amplicon makes the fluorescence increase of this system cumulative and exponential. The biased C_q_ value, resulting from this PCR efficiency dependent shift in the fluorescence increase, can be corrected with the C_shift_ factor, calculated as C_shift_ = log(1/(1-(1/E)))/log(E). This C_shift_ factor has previously been described for accumulation of fluorescence [6]. Despite the exponential character of fluorescence increase, an additional C_lag_ correction is required because of the appearance of fluorescence in only the 3rd or 4th cycle.

In summary, with respect to C_q_ corrections the chemistries can be distinguished into three categories: 1) the DNA-binding dyes, hydrolysis probes and LUX primers; 2) annealing probes and hairpin probes; and 3) QZyme probes. The three chemistries in the first category do not require correction of the observed C_q_ value to obtain the correct N_0_ value (Table 1). The second category requires a correction of -1 (dubbed C_lag_) to compensate for the fact that the fluorescence does not accumulate or is not observed until the next annealing phase. The third category, the QZyme system, requires besides the C_lag_ correction also an efficiency-dependent C_shift_ correction due to the nature of the increase in its fluorescence.

#### PCR efficiency

**Fig. 3:**
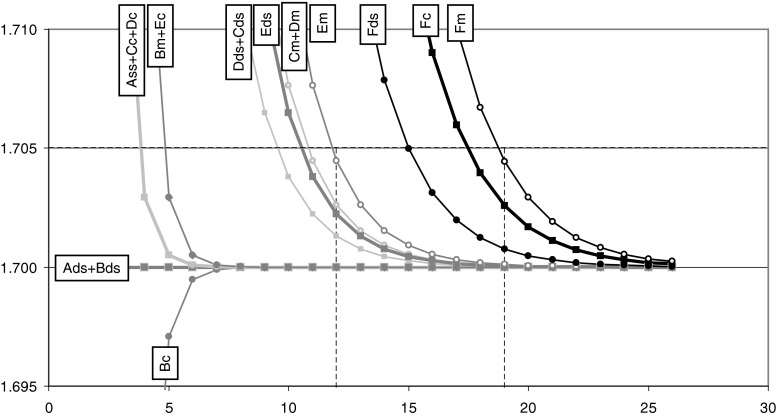
PCR efficiency values derived from the simulated amplification curves for all six chemistries, ds-DNA and ss-cDNA input and mRNA and cDNA targeting. Chemistry groups are identified by the character that was assigned in Fig. 1 with the suffixes ‘c’ and ‘m’ to indicate targeting of the cDNA or mRNA sequence for ss-cDNA input; a ds suffix indicates ds-DNA. The PCR efficiency was determined for windows of 4 data points starting at the cycle at which the value is plotted. Only efficiency values within 1 % of the valid efficiency value are displayed. An overestimation of 0.005 in efficiency value, in a range of C_q_ values of 15–25 would result in a 4–7 % underestimation of the target quantity. The vertical dotted lines indicate when the over-estimation of the PCR efficiency is below 0.005 for chemistry groups A through E, and group F, respectively. Note that the displayed PCR efficiency (1.7) represents the ‘worst case scenario’; for higher PCR efficiency values the curves become straight lines in earlier cycles (See Fig S10, ESM)

The PCR efficiency can be derived from the data points in the exponential phase of the amplification curve [4]. Although the deficits in the increase in fluorescence become negligible with increasing cycle number, the effect of this deficit on the slope of the line fitted through the data points remains significant. This results in an overestimation of the PCR efficiency (Fig. 3; Fig. S10, ESM). It can be calculated that, with C_q_ ranging from 15 to 25 cycles, a 0.005 overestimation of the PCR efficiency value, will result in a 4–7 % underestimation of the target quantity. When such an error is deemed acceptable, given other variation sources in qPCR [19], for most chemistries a sufficiently accurate PCR efficiency can be derived from the data points from cycle 12 and higher. However, for the QZyme system such a PCR efficiency value can only be derived after cycle 19. Of course, for all chemistries the PCR efficiency should be derived from data points in the exponential phase; when the above criterion is fulfilled there should still be at least four cycles before the plateau phase is reached.

## Discussion

In the current simulation of fluorescence increase for different qPCR monitoring chemistries, the effect of the type of DNA input, probe targeting and PCR efficiency were taken into account by counting the number of sense and anti-sense DNA strands and the number of fluorescence units associated with these strands in the generation of amplified DNA in every PCR cycle. This approach differs from the one used in our 2010 paper [6]. The accumulation of fluorescence of hydrolysis probes was then determined by summing up the fluorescence generated in previous cycles. The C_shift_ that was derived to correct for the pooled fluorescence could now be applied to the QZyme chemistry which displays such cumulative increase.

Assuming a PCR efficiency of 2, the current simulations show that the ‘cumulative’ fluorescence series of group C and D chemistries (1-3-7-15-31, being the cumulative of 1-2-4-8-16) is indistinguishable from the 2-4-8-16-32 series after the number of cycles that are in practice required to reach enough fluorescence to determine C_q_ (Figs. S2–S7 and Supplementary Excel file, ESM). Thus, DNA-binding dyes and hydrolysis probes display the ‘same’ amplification curve. However, for all chemistries, ss-DNA input will result in only half of the fluorescence compared to ds-DNA. This difference between ds-DNA input and ss-cDNA input is efficiency-dependent. C_q_ values observed for ds-DNA can be transformed into equivalent values for ss-cDNA input by adding a factor of log(2)/log(E).

The accumulation of fluorescence compensates the fluorescence deficit that most probe-based chemistries, irrespective of ds-DNA or ss-DNA input, suffer compared to DNA-binding dye fluorescence increase. Therefore, only annealing and hairpin probe systems (groups B and E), which are non-cumulative, require the C_lag_ correction. The latter correction is also required for those groups of chemistries in which fluorescence release starts in either the 2nd, 3rd or 4th cycle. However, the C_lag_ correction is still only -1 cycle for all these cases because, similar to the cumulative nature of hydrolysis probes, the exponential nature of these chemistries compensates for most of the lag.

Our calculations show that the initial differences in fluorescence accumulation when probes are targeting either the mRNA or cDNA sequences are negligible for the determination of C_q_ values and thus the calculation of DNA starting concentrations; qPCR assays using different probe orientations for the different genes can therefore be analyzed without having to account for the probe orientation.

Note that the derivation of the fluorescence increase kinetics is based on what theoretically should take place in the first cycles of the PCR reaction [20]. In fact it is unknown what really happens in those cycles. However, the mathematical truism that C_q_ is exponentially related to N_0_ means that a 1 cycle difference in C_q_ will result in an E-fold bias in the derived target quantity. To avoid this error the C_q_ value should be corrected for chemistry, input and targeting bias. However, this does also imply that the PCR efficiency should be determined correctly. A recent comparison of qPCR data analysis methods showed that approaches based on the analysis of amplification curves could perform as good, or better, than the commonly applied standard curve or dilution series method to determine the PCR efficiency [4]. Those amplification curve analysis methods derive an estimate of the PCR efficiency from the relative increase, or slope, of the curve of the observed fluorescence values. The current study shows that, despite the difference in the first cycles and the horizontal shift of the amplification curves, for all chemistries these curves are parallel on a logarithmic fluorescence axis (Figs. S8 and S9, ESM). As was previously shown for hydrolysis probes, an accurate PCR efficiency value can be derived from these curves [6]. The described correction of the observed C_q_ values with C_shift_ and C_lag_, depending on chemistry and input DNA, has been implemented in LinRegPCR (version 2013.1; http://LinRegPCR.nl).

## Electronic supplementary material

Below is the link to the electronic supplementary material.

ESM 1 (PDF 340 kb)

ESM 2 (XLS 424 kb)
