# Supplementary material for: Fluorescent-increase kinetics of different fluorescent reporters used for qPCR depend on monitoring chemistry, targeted sequence, type of DNA input and PCR efficiency
Source: Mikrochim Acta. 2014 Jan 14;181(13):1689–96. doi: 10.1007/s00604-013-1155-8 (PMC4167442; doi:10.1007/s00604-013-1155-8)
Supplement: Supplementary file 1 — (PDF 340 kb) [file 604_2013_1155_MOESM1_ESM.pdf]

Electronic Supplementary Material on

Fluorescent-increase kinetics of different fluorescent reporters used for qPCR depend on monitoring chemistry, targeted sequence, type of DNA input and PCR efficiency.

Jan M Ruijter, Peter Lorenz, Jari M Tuomi, Michael Hecker, Maurice JB van den Hoff

Supplementary Microsoft Excel file

Simulation of the creation of DNA strands and accompanying increase in fluorescence during PCR cycling for 6 categories of PCR-monitoring chemistries. The simulations consider ds-DNA or ss-cDNA as input as well as different directionality of the probes or primers involved in fluorescence generation. The PCR efficiency values can be modified (on sheet 'all_curves', cell B35) to observe its effect on C_q values and resulting target quantities. See the sheet 'Read_me' for further details.

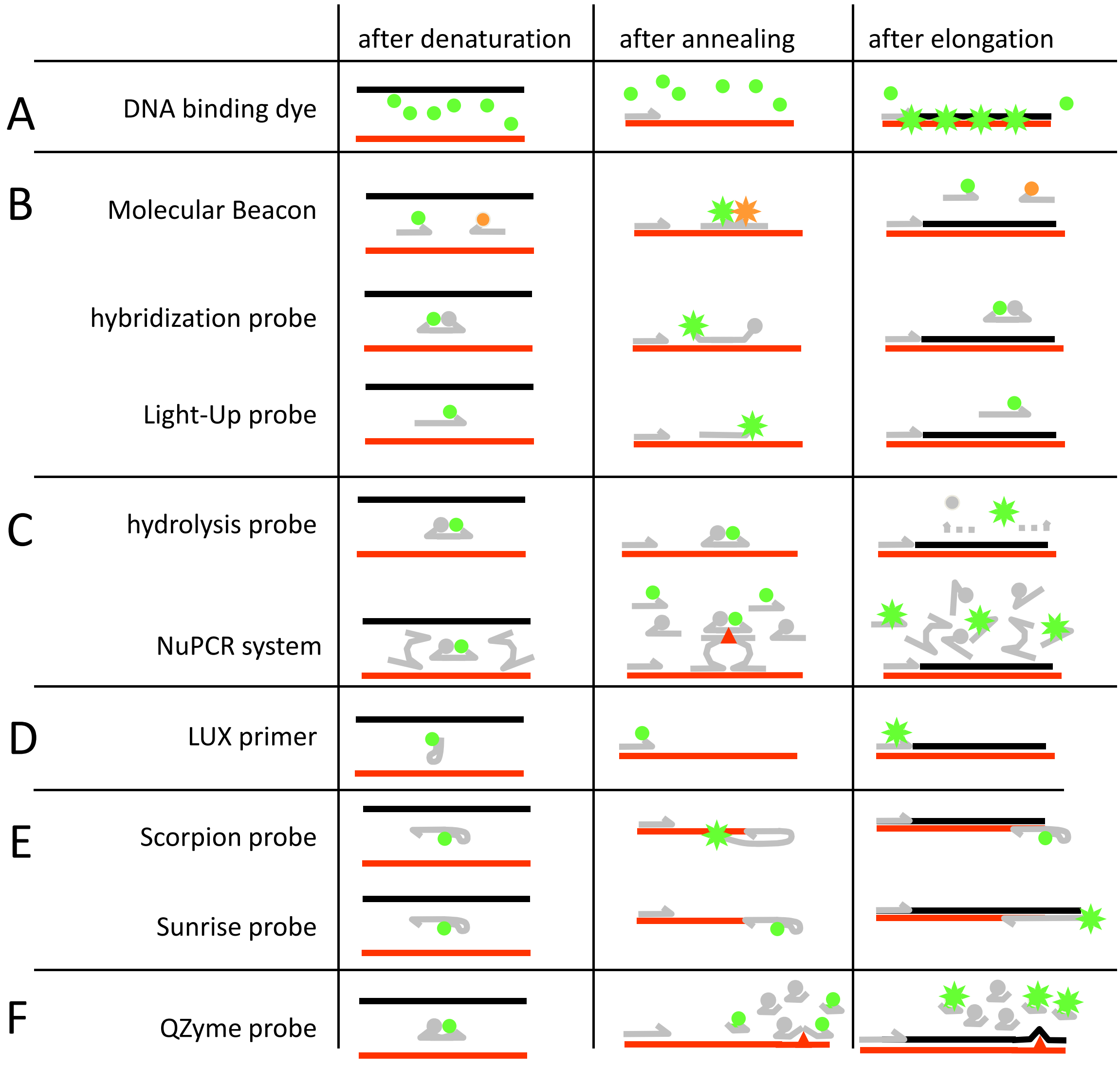

Figure S1. Comparison of the various monitoring chemistries discussed in this paper. For each chemistry group (**A-F**) the fluorescence status of the reporter or probe is illustrated after denaturation, annealing and elongation. B1, B2 and B3 represent hybridization probes, Molecular Beacons and Light-Up probes, respectively. These chemistries have been placed together in group B because they lead to equivalent fluorescence increase in all situations. For the same reason, hydrolysis probes and NuPCR are combined in group C and Scorpion and Sunrise probes in group E. **Red** strands represent the cDNA strand, black strands are synthesized complementary strands, primers and probes are shown as grey half arrows. **Grey** circles are quenchers; colored **circles** are quenched fluorophores whereas colored **stars** represent observed fluorescence emission. Red **triangles** indicate DNAzyme activity.

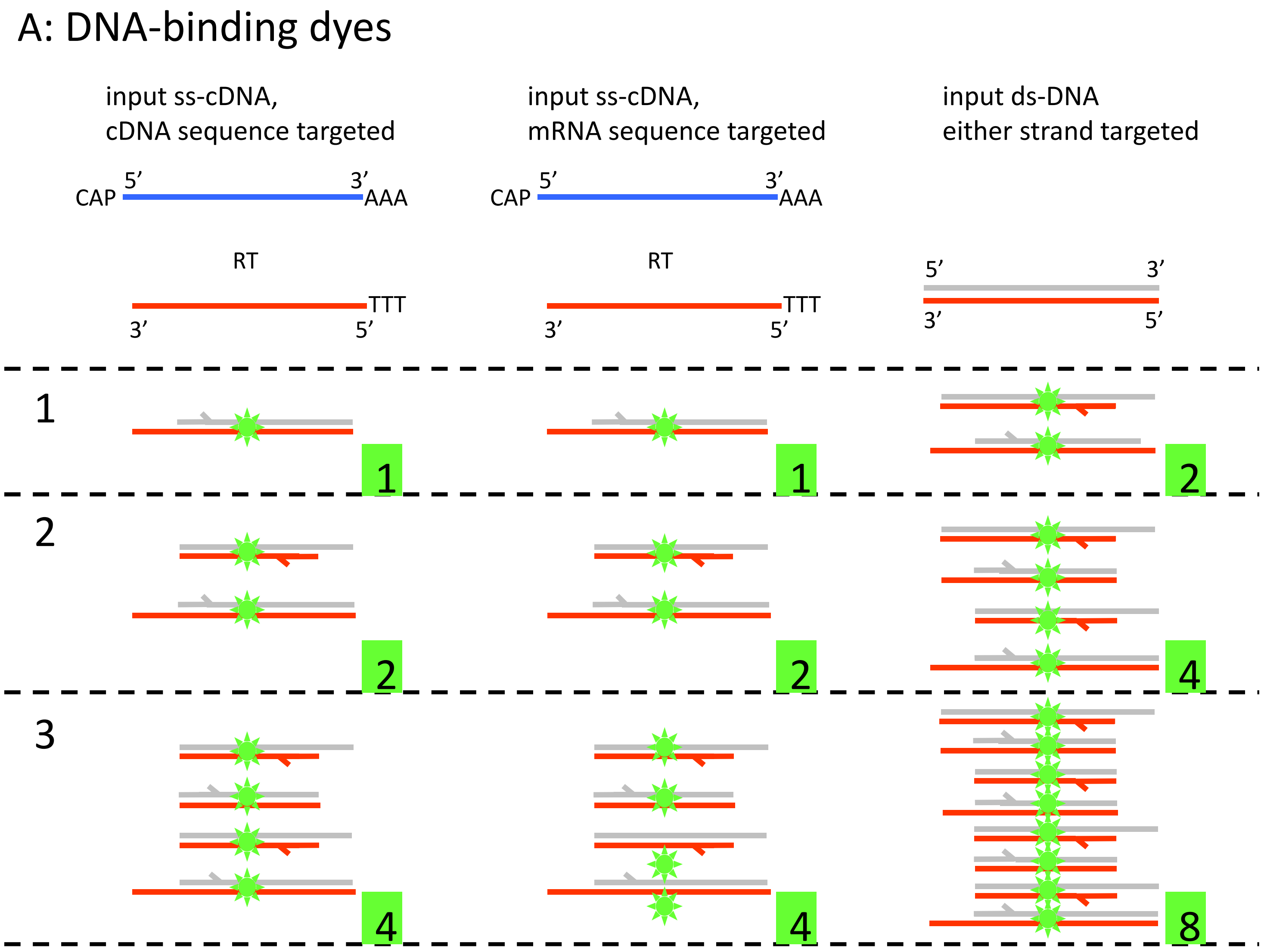

Figure S2 till S7. This series of figures displays cartoons of the fluorescence increase in the first PCR cycles for each of the groups of monitoring chemistries as defined in Fig. S1. Fluorescence increase was determined for input of ss-cDNA (after reverse transcription, **RT**) or ds-DNA (**right column**) and for probes targeting the cDNA sequence (**left**) or the mRNA sequence (**middle**) when the input is cDNA. For cDNA input, **red** strands represent the DNA strand present at start of the cycle; **grey** strands (including the primers indicated by an incorporated half arrow) are synthesized during the cycle. Horizontal dotted lines represent the annealing phase. Observed reporter fluorescence units are indicated by stars; (accumulated) fluorescence per cycle is given in the colored boxes. For each chemistry group and each input/targeting combination the fluorescence in the first 3 cycles (numbers at left) is given by a number at the start of the cycle (when fluorescence is maximal in the annealing phase) or a number at the end of the cycle (when fluorescence is maximal after elongation).

Figure S2. Group A: DNA-binding dyes. DNA-binding dyes are intercalating dyes that show increased fluorescence upon binding to ds-DNA [2]. Fluorescence returns to background level when the DNA melts into ss-DNA during denaturation. DNA-binding dyes display the standard exponential increase of fluorescence, following the mathematical pattern of 1-2-4-8 when the input is ss-cDNA and a 2-4-8-16 pattern when the input is ds-DNA.

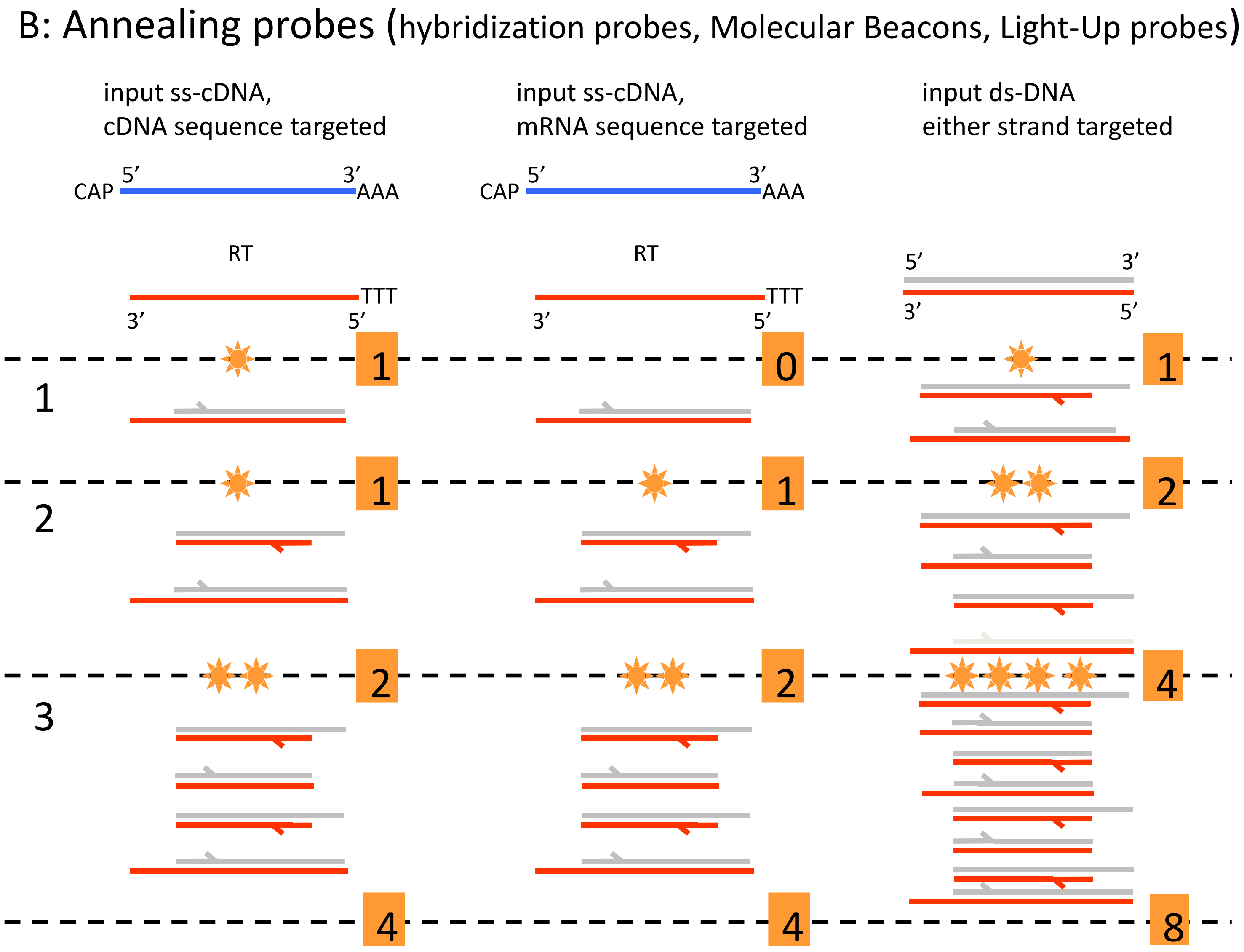

Figure S3. Group B: Annealing probes constitute three different chemistries (Hybridization Probes, Molecular Beacons and Light-Up probes) that display the same kinetic behavior. In Hybridization Probes [3] two fluorescently labeled probes have to anneal both to the same DNA strand to bring a donor fluorochrome in close proximity with an acceptor fluorochrome. The proximity allows FRET to occur and results in emission of light with a shorter wavelength. For Molecular Beacons [4] the situation is reversed. In free beacons, a hairpin-loop keeps the reporter and quencher close together. After annealing the hairpin opens up and the reporter efficiently emits fluorescence. Light-Up probes are peptide nucleic acid (PNA) oligonucleotides to which a thiazole dye is tethered. Probe annealing allows the dye to interact with the probe-DNA hybrid and to become brightly fluorescent [5]. The fluorescence of annealing probes is measured during the annealing phase. During elongation all three kinds of annealing probes are dissociated unscathed and their fluorescence returns to background levels. When the annealing probe targets the anti-sense strand (cDNA sequence; i.e. probe in sense-orientation), the targeted sequence is present at the start of the first cycle and the increase in fluorescence follows a 1-2-4-8 series with ds-DNA input and a 1-1-2-4 series with ss-cDNA input. When the probe targets the mRNA strand, the increase in fluorescence lags one cycle behind, following the 0-1-2-4 series.

C: Hydrolysis probes (including NuPCR)

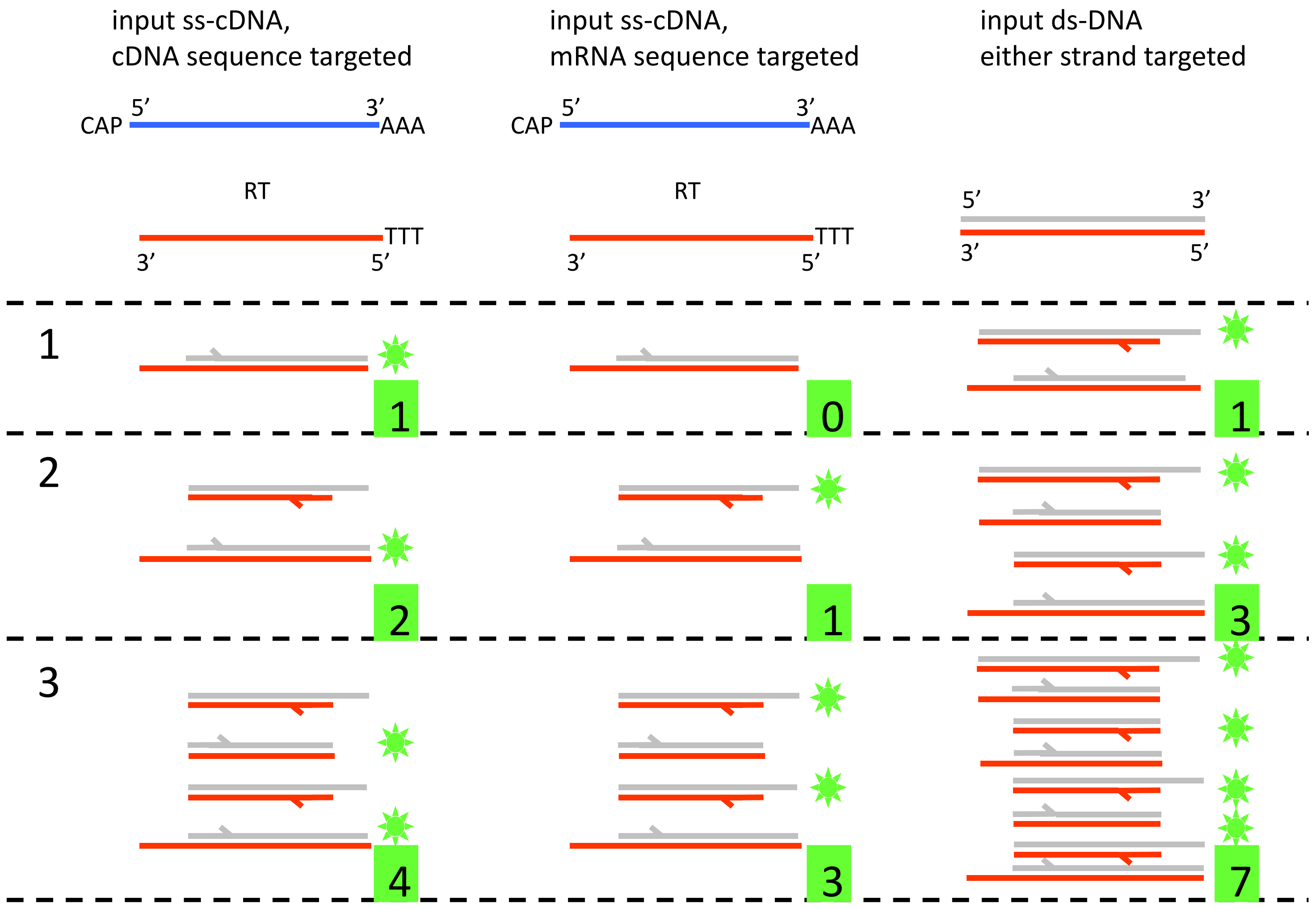

Figure S4. Group C: Hydrolysis probes constitute two chemistries: hydrolysis probes and NuPCR. Hydrolysis probes consist of probe sequences with a reporter and a quencher in close proximity, which ensures low background fluorescence in the unbound and annealed probes. During elongation the annealed probe is digested by the polymerase and the quenching is abolished. The released reporter is, and remains, fluorescent in all subsequent cycles, thus leading to accumulation of fluorescence [1]. The NuPCR system (Illumina Inc.) [7], consists of a combination of the annealing and hydrolysis probes in which two immediately adjacent probes bind to the target sequence and thus bring together two halves of a DNAzyme (called NuZyme). This DNAzyme then repeatedly binds and cleaves a universal hydrolysis probe which results in amplification of fluorescent signal. Because of the fixed length of the annealing phase the kinetics of the fluorescence increase follows the pattern of a hydrolysis probe. In case of ds-DNA input, both DNA strands are present and as a consequence the polarity of the probe does not affect the fluorescence increase which follows the standard cumulative series: 1-3-7-15. However, in case of ss-cDNA input, the fluorescence that is released depends on the polarity of the probe and can be either 0-1-3-7, when the mRNA strand is targeted or 1-2-4-8 when the cDNA strand is targeted.

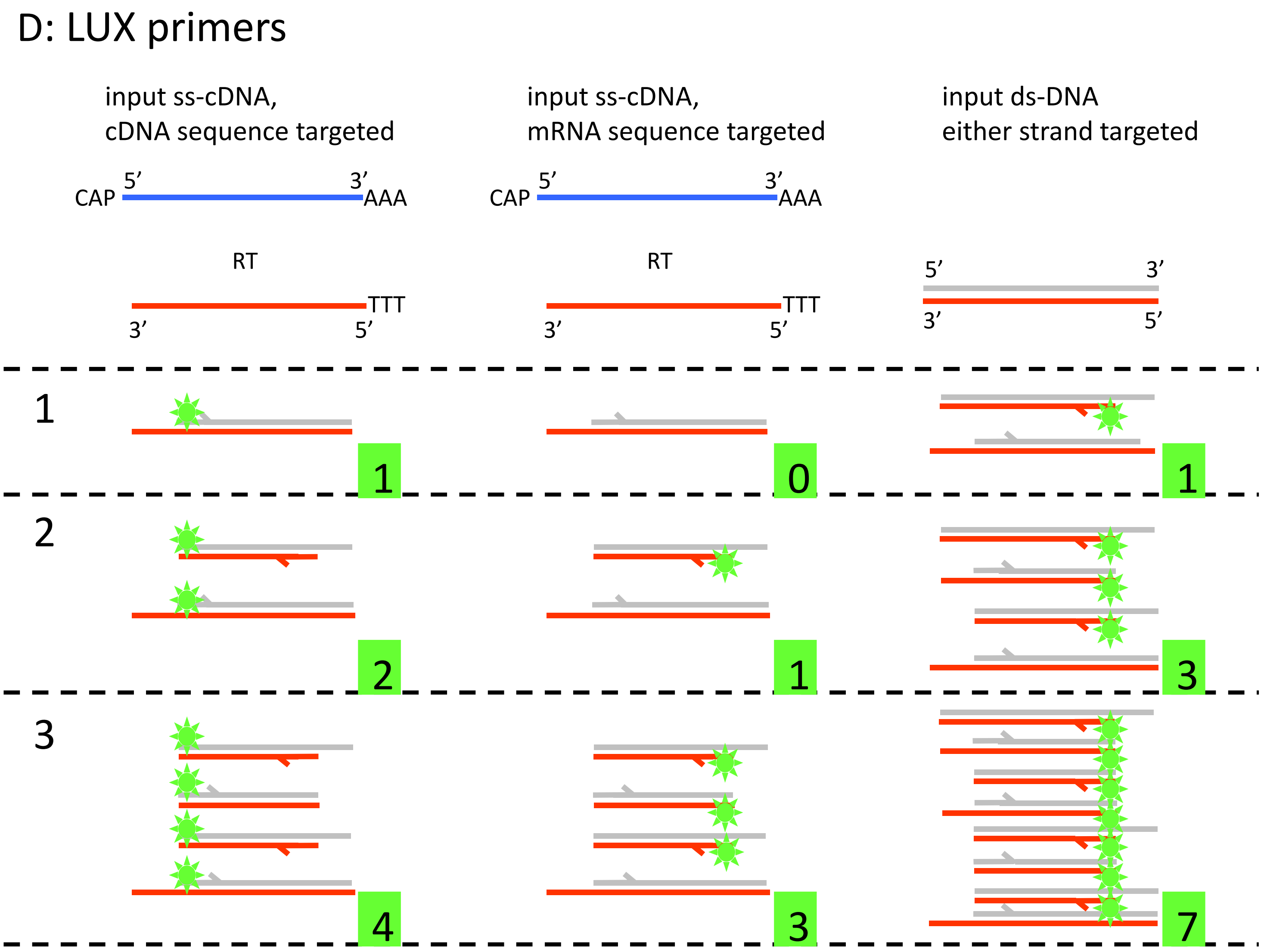

Figure S5. Group D: LUX primers. In the Light-Upon-eXtension (LUX) monitoring chemistry [8,9], one of the PCR primers is tagged with a fluorescent reporter within a hairpin structure that quenches the reporter. During annealing the hairpin opens and the reporter becomes fluorescent. After elongation the primer, and thus the reporter, is incorporated into one strand of the amplification product. Consequently, the reporter remains fluorescent during all subsequent PCR cycles and shows a cumulative behavior. In case of ss-cDNA input and a LUX primer that targets the cDNA sequence, the fluorescence increase follows a 1-2-4-8 series. However, when the primer targets the mRNA sequence no fluorescent primer is incorporated in the first cycle and this deficit propagates resulting in a 0-1-3-7 series. When the input is ds-DNA the targeted strand is always present resulting in the 1-3-7-15 series. The kinetics of fluorescence increase of LUX primers is thus the same as those of hydrolysis probes.

E: Hairpin primers / probes (Scorpion and Sunrise)

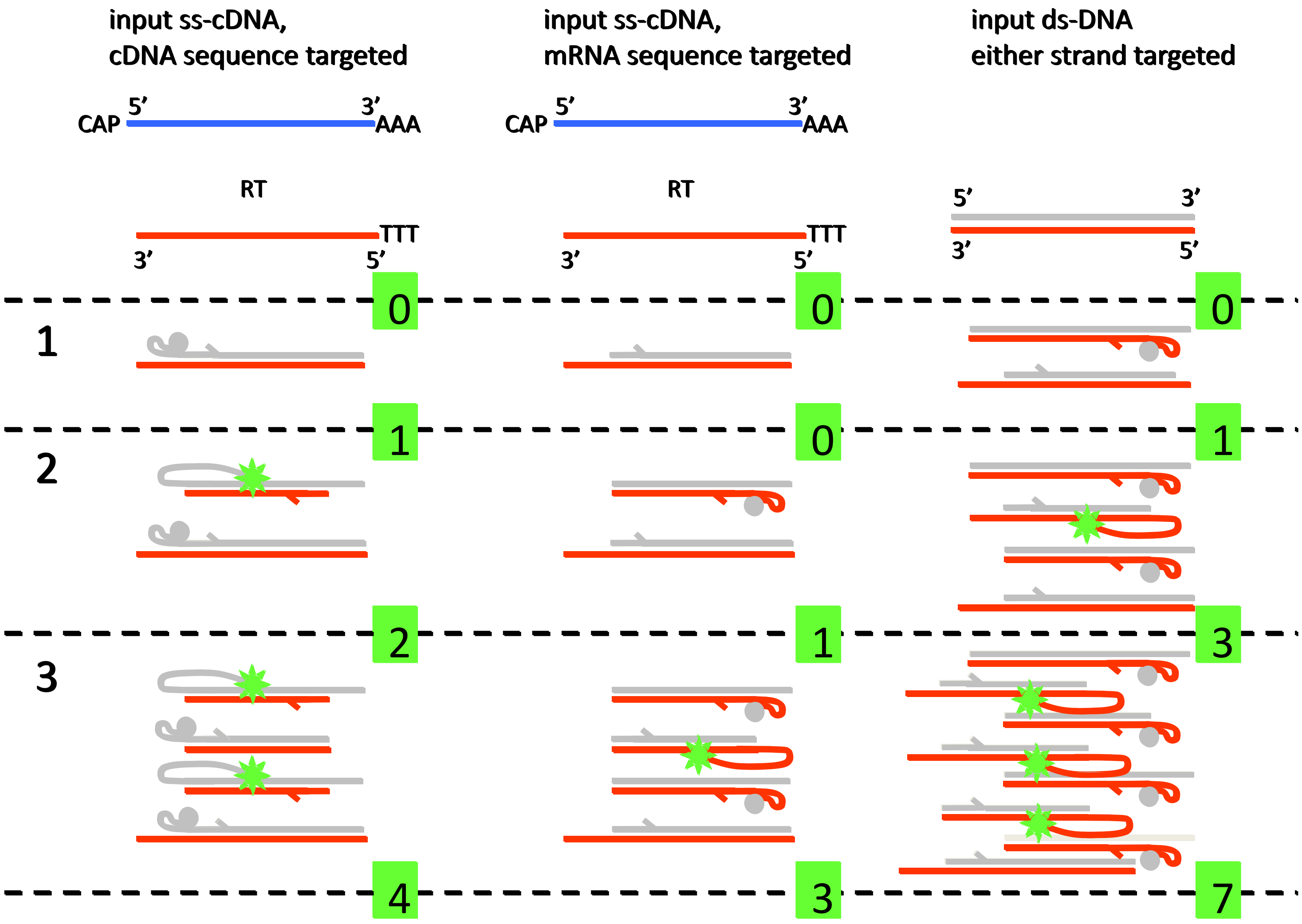

Figure S6. Group E: Hairpin probes: Scorpion probes and Sunrise probes. Scorpion primers [10] are a combination of a molecular beacon and a primer, separated by a sequence that prevents read-through by the polymerase. Additionally, in Scorpion primers, the hairpin loop comprises a sequence complementary to the amplicon sequence. During the annealing phase the Scorpion primer will not only hybridize to the respective end of the amplicon but also to the amplicon sequence complimentary to the hairpin. As a result of the latter hybridization the fluorescent reporter and quencher are physically separated allowing the reporter to fluoresce. During subsequent elongation the hybridized hairpin sequence is displaced allowing the Scorpion primer to form the hairpin structure again, due to which the quencher interacts with the reporter and inhibits fluorescence. Because the primer and the hairpin are incorporated in the amplicon, the Scorpion reporter will fluoresce again in each subsequent annealing step. Sunrise probes [11] are similar to Scorpion probes but they do lack the sequence complementary to the amplicon in the hairpin loop, as well as the read-through prevention. Sunrise primers are incorporated in the first cycle and the hairpin sequence becomes double stranded in the second cycle separating the quencher and the reporter allowing its fluorescence in all PCR cycles. Although Scorpion and Sunrise primers differ in structure, they show similar kinetics of fluorescence increase. When the reporter-containing primer targets the cDNA sequence and the input is ss-cDNA, 1 quenched reporter unit is incorporated in the first cycle which only starts to fluoresce in the annealing phase of the second cycle leading to a fluorescence increase series of 0-1-2-4-8. However, when the mRNA sequence is targeted, the first quenched reporter is incorporated in the second cycle to become fluorescent in the third cycle when also 2 quenched units are incorporated. This results in a fluorescence series of 0-0-1-3-7. In case of ds-DNA input both strands are available and the incorporation of quenched units follows a 1-2-4-8 series resulting in a fluorescence increase series of 0-1-3-7-15.

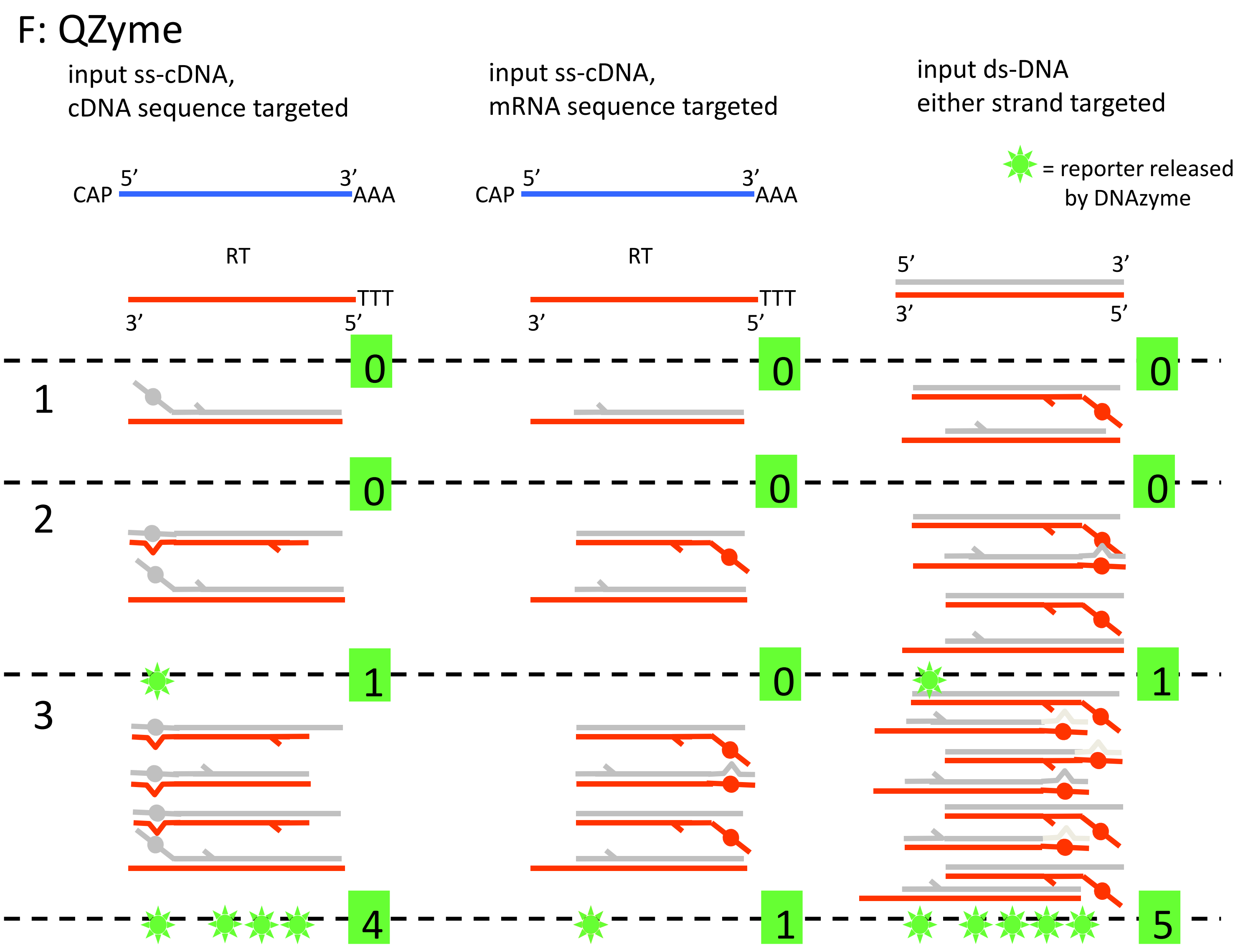

Figure S7. Group F: QZyme. The QZyme system [12] comprises two primers and a quenched reporter substrate. One of the primers is a normal gene-specific primer and the other one (dubbed QZyme-primer) is also gene-specific, but carries a 5'-extension of a DNAzyme sequence in its inactive anti-sense orientation. Synthesis of the complementary strand during the extension phase of a PCR cycle produces the active QZyme sense strand. During the annealing phase of the following cycle the active QZyme repeatedly binds and cleaves the reporter substrate, thus abolishing quencher activity and releasing fluorescence. This shift in fluorescence generation of a newly generated QZyme into the next PCR cycle leads to a fluorescence increase series that starts with two or three lag cycles followed by an increase in fluorescence following a cumulative 1-4-11 series, for ss-cDNA input and a QZyme primer targeting the cDNA sequence. A cumulative 1-5-16 series is observed in case of either ds-DNA input or ss-cDNA input and a QZyme primer targeting the mRNA sequence. Because the DNAzyme is activated in every annealing phase the fluorescence increase is cumulative and exponential.

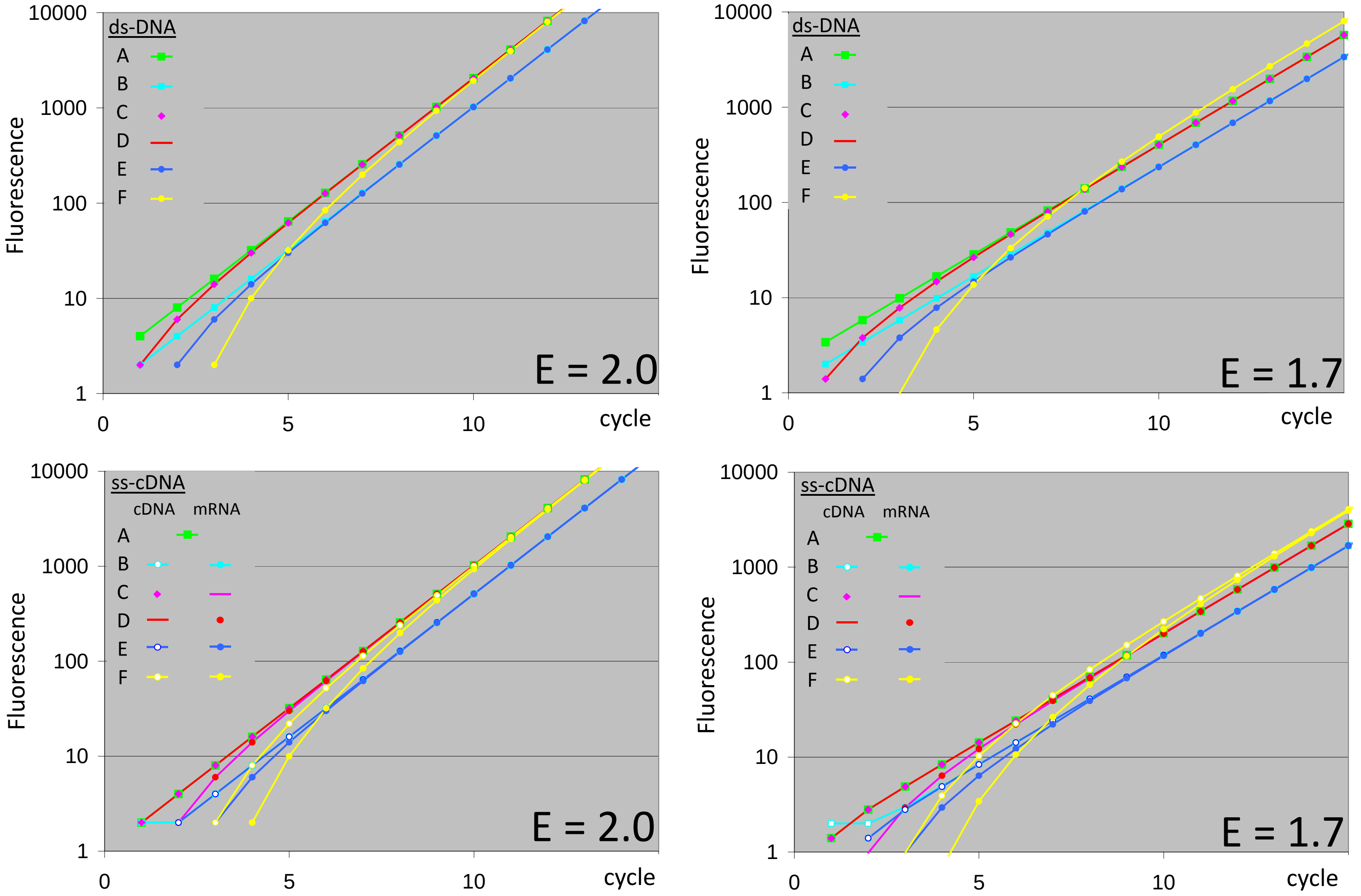

Figure S8. Amplification curves for all 6 chemistries were simulated for PCR efficiency of 2.0 (left) and 1.7 (right) and input of ds-DNA (top) and ss-cDNA (bottom) plotted on a logarithmic fluorescence axis. Chemistry groups are identified by the character that was assigned in Fig. 1. The first 20 cycles are shown to illustrate the differences in initial increase of fluorescence between chemistries. Despite those initial differences, the amplification curves converge into groups of overlapping parallel straight lines for both PCR efficiencies.

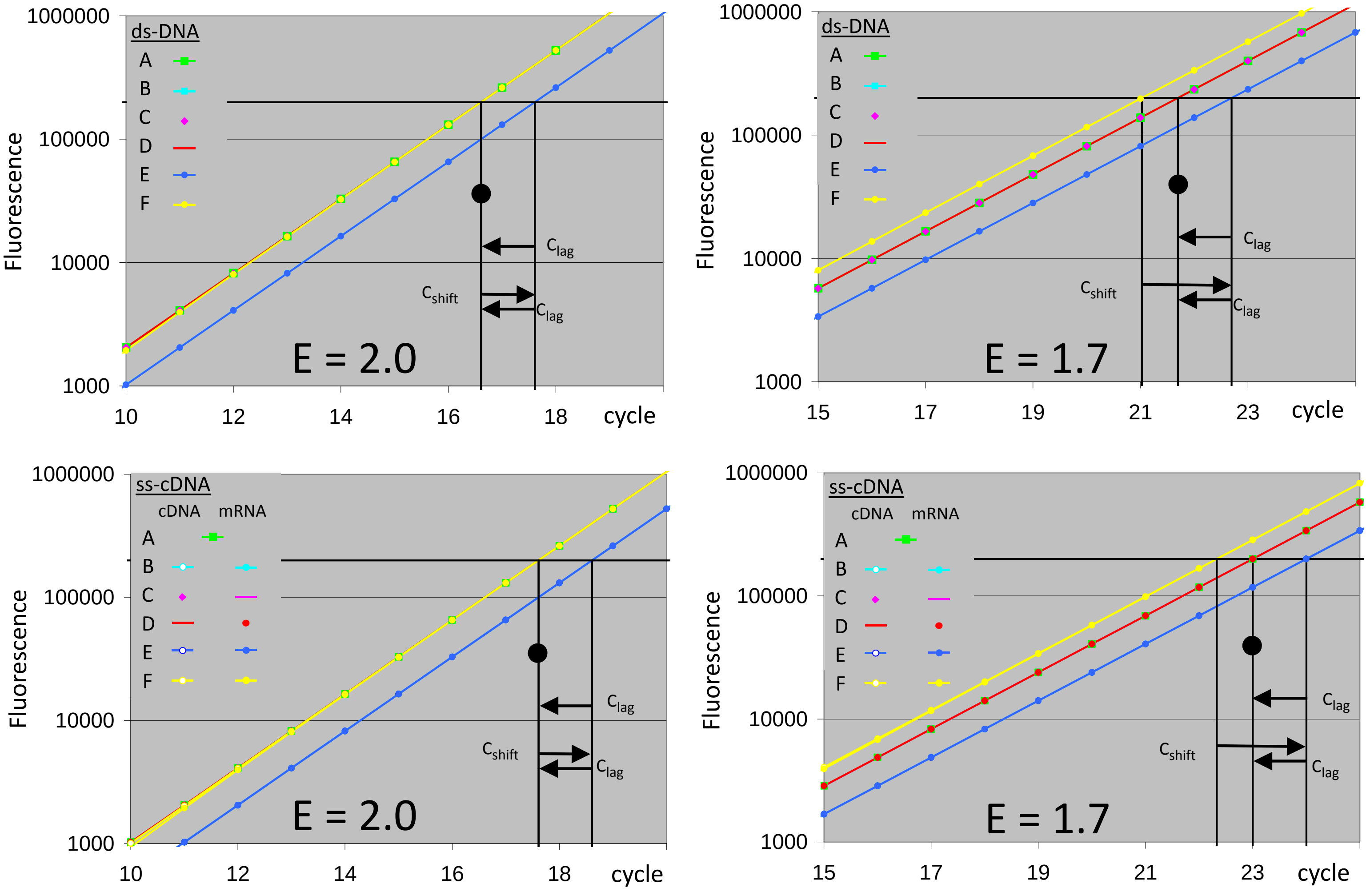

Figure S9. Amplification curves for all 6 chemistries were simulated for PCR efficiency of 2.0 (left) and 1.7 (right) and input of ds-DNA (top) and ss-cDNA (bottom) plotted on a logarithmic fluorescence axis. Chemistry groups are identified by the character that was assigned in Fig. 1. The cycles around the C_q values are shown. Note that depending on the chemistry and the PCR efficiency C_q values are shifted with respect to the C_q that is observed with a DNA-binding dye (black circle). Chemistries in groups A, C and D do not require a C_q correction (●); chemistries in groups B and E require a C_{lag} correction (🡐) and the chemistry in group F requires a C_{shift} correction (🡒) and a C_{lag} (🡐) correction. Note that these two corrections cancel each other out when the PCR efficiency is 2 (left).

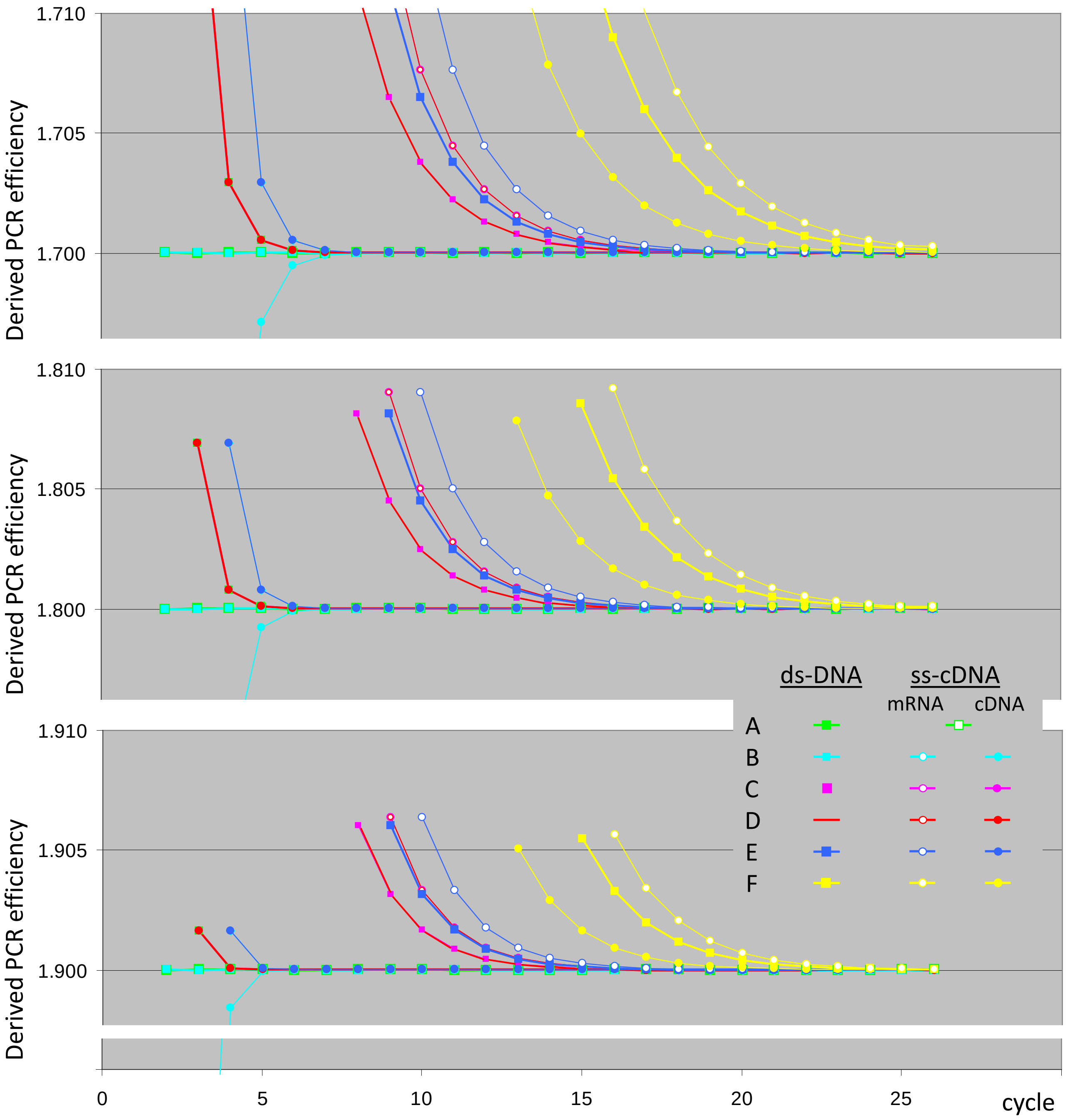

Figure S10. PCR efficiency values derived from the simulated amplification curves for all 6 chemistries, ds-DNA and ss-cDNA input and mRNA and cDNA targeting. Chemistry groups are identified by the character that was assigned in Fig. 1. The PCR efficiency was determined for windows of 4 data points starting at the cycle at which the value is plotted. Only efficiency values within 1% of the valid efficiency value are displayed. An overestimation of 0.005 in efficiency value, in a range of C_q values of 15 – 25 would result in a 4-7% underestimation of the target quantity.

References

1. Tuomi JM, Voorbraak F, Jones DL, Ruijter JM (2010) Bias in the Cq value observed with hydrolysis probe based quantitative PCR can be corrected with the estimated PCR efficiency value. Methods 50: 313-322.
2. Wittwer CT, Herrmann MG, Moss AA, Rasmussen RP (1997) Continuous fluorescence monitoring of rapid cycle DNA amplification. BioTechniques 22: 130-138.
3. Marras SA (2008) Interactive fluorophore and quencher pairs for labeling fluorescent nucleic acid hybridization probes. Mol Biotechnol 38: 247-255.
4. Tyagi S, Kramer FR (1996) Molecular beacons: probes that fluoresce upon hybridization. Nat Biotechnol 14: 303-308.
5. Svanvik N, Westman G, Wang D, Kubista M (2000) Light-up probes: thiazole orange-conjugated peptide nucleic acid for detection of target nucleic acid in homogeneous solution. Anal Biochem 281: 26-35.
6. Holland PM, Abramson RD, Watson R, Gelfand DH (1991) Detection of specific polymerase chain reaction product by utilizing the 5'----3' exonuclease activity of Thermus aquaticus DNA polymerase. Proc Natl Acad Sci U S A 88: 7276-7280.
7. Mokany E, Tan YL, Bone SM, Fuery CJ, Todd AV (2013) MNAzyme qPCR with superior multiplexing capacity. Clin Chem 59: 419-426.
8. Lowe B, Avila HA, Bloom FR, Gleeson M, Kusser W (2003) Quantitation of gene expression in neural precursors by reverse-transcription polymerase chain reaction using self-quenched, fluorogenic primers. Anal Biochem 315: 95-105.
9. Nazarenko I, Lowe B, Darfler M, Ikonomi P, Schuster D, Rashtchian A (2002) Multiplex quantitative PCR using self-quenched primers labeled with a single fluorophore. Nucleic Acids Res 30: e37.
10. Whitcombe D, Theaker J, Guy SP, Brown T, Little S (1999) Detection of PCR products using self-probing amplicons and fluorescence. Nat Biotechnol 17: 804-807.
11. Nazarenko IA, Bhatnagar SK, Hohman RJ (1997) A closed tube format for amplification and detection of DNA based on energy transfer. Nucleic Acids Res 25: 2516-2521.
12. Mokany E, Todd AV, Fuery CJ, Applegate TL (2006) Diagnosis and monitoring of PML-RARalpha-positive acute promyelocytic leukemia by quantitative RT-PCR. Methods Mol Med 125: 127-147.
